# Preliminary study of proton magnetic resonance spectroscopy to assess bone marrow adiposity in the third metacarpus or metatarsus in Thoroughbred racehorses

**DOI:** 10.1111/evj.14086

**Published:** 2024-05-03

**Authors:** Charlotte L. Hewitt‐Dedman, Lucy E. Kershaw, Tobias Schwarz, Jorge Del‐Pozo, Juliet Duncan, Carola R. Daniel, Eugenio Cillán‐García, Maria Chiara Pressanto, Sarah E. Taylor

**Affiliations:** ^1^ Royal (Dick) School of Veterinary Studies and Roslin Institute The University of Edinburgh Roslin UK; ^2^ BHF Centre for Cardiovascular Science and Edinburgh Imaging University of Edinburgh Edinburgh UK

**Keywords:** bone marrow adiposity, bone mineral density, computed tomography, horse, magnetic resonance imaging

## Abstract

**Background:**

Magnetic resonance spectroscopy (MRS) has been used to investigate metabolic changes within human bone. It may be possible to use MRS to investigate bone metabolism and fracture risk in the distal third metacarpal/tarsal bone (MC/MTIII) in racehorses.

**Objectives:**

To determine the feasibility of using MRS as a quantitative imaging technique in equine bone by using the ^1^H spectra for the MC/MTIII to calculate fat content (FC).

**Study design:**

Observational cross‐sectional study.

**Methods:**

Limbs from Thoroughbred racehorses were collected from horses that died or were subjected to euthanasia on racecourses. Each limb underwent magnetic resonance imaging (MRI) at 3 T followed by single‐voxel MRS at three regions of interest (ROI) within MC/MTIII (lateral condyle, medial condyle, proximal bone marrow [PBM]). Percentage FC was calculated at each ROI. Each limb underwent computed tomography (CT) and bone mineral density (BMD) was calculated for the same ROIs. All MR and CT images were graded for sclerosis. Histology slides were graded for sclerosis and proximal marrow space was calculated. Pearson or Spearman correlations were used to assess the relationship between BMD, FC and marrow space. Kruskal–Wallis tests were used to check for differences between sclerosis groups for BMD or FC.

**Results:**

Eighteen limbs from 10 horses were included. A negative correlation was identified for mean BMD and FC for the lateral condyle (correlation coefficient = −0.60, *p* = 0.01) and PBM (correlation coefficient = −0.5, *p* = 0.04). There was a significant difference between median BMD for different sclerosis grades in the condyles on both MRI and CT. A significant difference in FC was identified between sclerosis groups in the lateral condyle on MRI and CT.

**Main limitations:**

Small sample size.

**Conclusions:**

^1^H Proton MRS is feasible in the equine MC/MTIII. Further work is required to evaluate the use of this technique to predict fracture risk in racehorses.

## 1 INTRODUCTION

Fractures of MC/MTIII are one of the most common injuries in Thoroughbred racehorses in the United Kingdom and have been associated with a high number of fatalities during National Hunt racing.^1^ These injuries are a significant welfare concern within the industry, therefore the hope is that continued investigation into a variety of advanced imaging techniques will provide further information on the pathophysiology of bone structure and modelling responses within the distal metacarpal or metatarsal condyle with the ultimate aim of reducing the prevalence of catastrophic fractures.^2^, ^3^, ^4^

Many advanced imaging studies have been used to assess the third metacarpal/tarsal bone however these imaging modalities cannot currently be used to reliably identify all horses at risk of sustaining a complete fracture of the metacarpal/tarsal condyle. Several parameters were assessed as screening tools in one study using high‐resolution peripheral quantitative CT, however these were found to have limited ability to identify horses at increased risk of fracture.^5^ Recently, positron emission tomography using ^18^F‐sodium fluoride has been shown to be useful for the detection of stress remodelling in the metacarpo/tarsophalageal joints of Thoroughbred racehorses. However, continued investigation is required to define normal ranges for maximal standardised uptake values and assess the relationship with clinical outcome or potential catastrophic injury.^6^, ^7^, ^8^ A recent cadaver study looked at MRI‐detectable pre‐fracture markers using a 1.5 T MRI system and found an increased depth of dense subchondral/trabecular bone in the palmar half of the lateral parasagittal groove of the distal third metacarpus in horses that had sustained a fracture, however the prevalence of fractures in the cadaver groups studied was far higher than that encountered during racing and training and it is questionable if this technique could perform accurately at the low prevalences observed.^4^

Magnetic resonance spectroscopy (MRS) is a non‐invasive quantitative advanced imaging technique which can be used alongside MRI to investigate metabolic changes within bone by quantifying bone marrow fat content (FC).^9^, ^10^, ^11^ Bone marrow adiposity has been found to increase with decreasing bone mineral density and this technique has been investigated in humans with a variety of metabolic diseases such as osteoporosis to determine fracture risk, as well as in high level athletes to assess changes in marrow fat composition.^11^, ^12^, ^13^, ^14^, ^15^, ^16^

Magnetic resonance spectroscopy has identified a decrease in bone marrow adiposity in the lumbar spine and femoral neck in professional football players compared with controls^15^ and a similar finding was reported in runners.^16^ Additionally, a group of 24 women on bed rest for 60 days, were found to have an increase in bone marrow adiposity that persisted for up to a year after the bed rest.^17^ This evidence suggests that changes in bone marrow adiposity, detected by MRS are seen in response to altered exercise in humans and a similar change may be observed in horses in training. To date, MRS has not been performed in equine bone and it is not known whether a spectrum of ^1^H protons can be obtained. Magnetic resonance spectroscopy of bone can be challenging due to the low water and fat content leading to poor signal. This could be particularly difficult in the dense condyles of the third MC/MTIII.

The aim of this study was to determine the feasibility of using MRS as a quantitative imaging technique in equine bone by using the ^1^H spectra for the equine MC/MTIII to calculate FC. Our hypotheses were: (1) MRS is a useful technique to quantify bone marrow FC in equine bone, and (2) there is a relationship between FC and BMD in both condyles of the third metacarpus/tarsus in the trained racehorse but not in the proximal bone marrow of the distal diaphysis of the third metacarpus/tarsus.

## 2 MATERIALS AND METHODS

### 2.1 Sample collection

A convenience sample of limbs from Thoroughbred racehorses in training was collected from horses that died or were subjected to euthanasia on Northern English and Scottish racecourses between September 2019 and September 2021. The limbs were frozen in a −20° freezer within 24 h following euthanasia.

### 2.2 MRI and MRS

**FIGURE 1:**
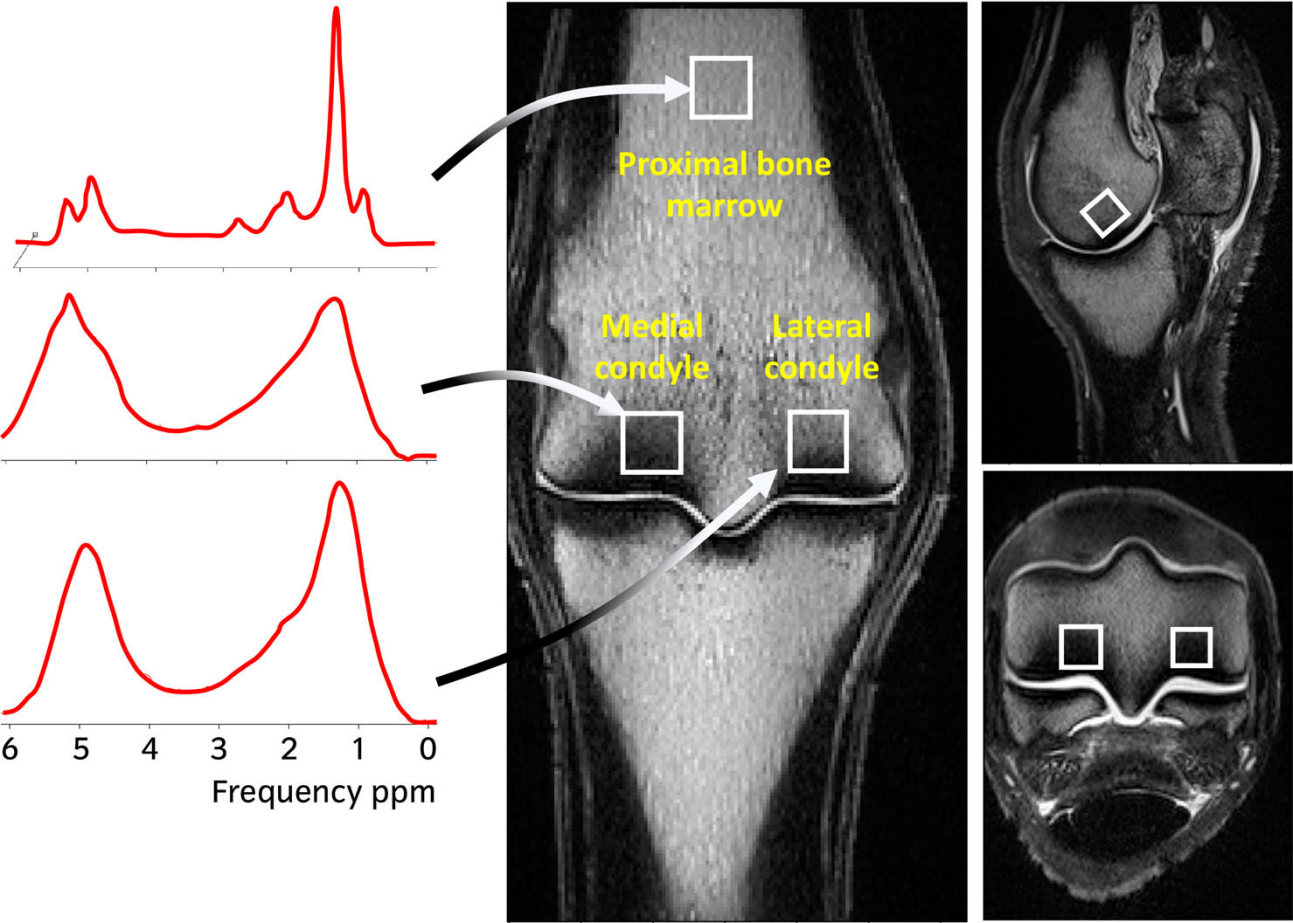
T1‐weighted images showing magnetic resonance spectroscopy ROI positioning and example spectra for each of the ROIs. The ROIs for the lateral and medial condyles are 5 mm proximal to the distal articular margin of MC/MTIII with the axial border of the ROI at the level of the lateral or medial parasagittal groove. The ROI for the proximal bone marrow of the third metacarpus/tarsus is in the centre of the distal diaphysis of MC/MTIII, 7 cm proximal to the distal aspect of the sagittal ridge. The spectra are made up of different resonance peaks based on the chemical shift properties of protons within the region of interest in the bone. Each of these peaks appear at known chemical shifts which allow them to be identified as triglycerides or water. The frequency is shown in parts per million (ppm) along the x‐axis.

The limbs were defrosted at room temperature for 24 h prior to scanning. Radiographs were taken to check for metal; limbs with metallic implants were excluded. Magnetic resonance scans were acquired on a high‐field (3 T) MRI System (MAGNETOM Skyra, Siemens Healthineers) using a knee coil. The following sequences were obtained in three orthogonal planes: T1 weighted (T1W) 3D turbo spin echo (TSE), T2*weighted (T2*W) 3D gradient echo (GRE), T2*‐weighted gradient spoiled multiple echo data image combination (MEDIC) and turbo inversion recovery magnitude (TIRM) for fat suppression. MRS was performed using a Stimulated Echo Acquisition Mode (STEAM) sequence (Figure S1). The MRS measurement region of interest (ROI) (1 cm^3^) was placed at three sites; the lateral condyle (5 mm proximal to the distal articular margin of MC/MTIII in the axial portion of the palmar third of the condyle including the subchondral bone and subjacent trabecular bone), the medial condyle (5 mm proximal to the distal articular margin of MC/MTIII in the axial portion of the palmar third of the condyle including the subchondral bone and subjacent trabecular bone) and in the centre of the distal diaphysis of MC/MTIII, 7 cm proximal to the distal aspect of the sagittal ridge (proximal bone marrow or PBM) (Figure 1).

The MR images were independently evaluated and graded by three experienced clinicians (an ECVS Specialist in Equine Surgery, an ECVS resident and an ECDVI Specialist in Diagnostic imaging). The reviewers were blinded to the identification and history of the horse throughout the study. The lateral and medial condyles of MC/MTIII were graded for sclerosis from grade 0 (normal) to grade 3 (severe) as described in previous studies^18^, ^19^ (Figure S1). Sclerosis was defined as an area of reduced signal intensity on all sequences. Each reviewer recorded their grades independently and if there was disagreement in the grades allocated then a consensus grade was agreed upon.

Spectra were analysed in JMRUI^20^ using AMARES,^21^ fitting the water peak and 6 fat peaks. The amplitude of the fat and water peaks were used to calculate percentage FC for each ROI as previously described.^11^

### 2.3 Quantitative CT

Computed tomography scans were acquired on a helical 64‐slice CT scanner (Somatom Definition AS, Siemens Healthineers) (Figure S2). The osteodensitometry phantom was placed under all of the cadaver limbs. It contained two chambers; one containing demineralised water with a reference value of 0 Hounsfield Units (HU) and one containing calcium hydroxyapatite (CaHAP) with a concentration of 200 mg/mL.

The CT images were independently evaluated by the same clinicians that graded the MR images and each condyle of MC/MTIII was given a sclerosis grade from 0 (normal) to 3 (severe) adapted from a previous study^18^ (Figure S2). Sclerosis was defined as a region of hyperattenuation within condyles. Each reviewer recorded their grades independently and if there was disagreement in the grades allocated then a consensus grade was agreed upon.

Mean bone mineral density (BMD) was calculated for the lateral condyle (5 mm proximal to the distal articular margin of MC/MTIII in the axial portion of the palmar third of the condyle including the subchondral bone and subjacent trabecular bone), medial condyle (5 mm proximal to the distal articular margin of MC/MTIII in the axial portion of the palmar third of the condyle including the subchondral bone and subjacent trabecular bone) and the PBM (7 cm proximal to the distal aspect of the sagittal ridge) using the following formula^22^:

$$\text{Mean}\;\mathrm{BMD}=200\;\mathrm{mg}/\mathrm{mL}\frac{\mathrm{HUt}}{\mathrm{HUb}-\mathrm{HUw}},$$

HUt: measured density of bone (Hounsfield Units [HU]); HUb: measured density of CaHAP solution of phantom (HU); HUw: measured density of demineralised water of phantom.

### 2.4 Histopathology

The MC/MTIII was harvested. A 2 mm slice of bone was taken from the distal diaphysis of the bone in a transverse plane, 7 cm proximal to the distal aspect of the sagittal ridge. The slices were matched as best as possible to the centre of the ROI used for MRS and CT. Two millimetre slices of bone were taken from the lateral and the medial parasagittal grooves and the middle of the lateral and medial condyles in a sagittal plane. These slices of bone were placed in a decalcifying solution (Leica Surgipath Decalcifier 1 Solution) for 4 weeks and were then processed on the Epredia Excelsior AS Tissue processor. These sections were then embedded in wax, cut at a thickness of 4 μm and stained by the routine Haematoxylin and Eosin method.

The histology slides were independently evaluated by three clinicians (a ECVS Specialist in Equine Surgery, a ECVS resident and a ECVP Specialist in Veterinary Pathology) and each condyle was graded for sclerosis of the subchondral bone plate and adjacent cancellous bone from 0 (normal) to 3 (severe) as previously described^23^ (Figure S3). Each reviewer recorded their grades independently and if there was disagreement in the grades allocated then a consensus grade was agreed upon.

The percentage marrow space was calculated as a proxy for FC from ROIs in the lateral condyle, medial condyle and the centre of the distal diaphysis of each limb using QuPath digital pathology software.^24^

### 2.5 Data analysis

Data were analysed and visualised using R version 4.1.1 (RStudio PBC).^25^ Descriptive statistics were calculated. Shapiro–Wilk tests were used to test for normality. Where data were not normally distributed, the median was calculated. Interclass Correlation Coefficient (ICC) estimates were calculated for MRI, CT and histology sclerosis grade based on a mean‐rating (*k* = 3), absolute‐agreement, two‐way mixed‐effects model. Pearson or Spearman correlations were used to assess the relationship between BMD from CT, FC from MRS and marrow space from histology for each ROI. Kruskal–Wallis tests were used to check for differences between sclerosis groups for BMD or FC. A *p*‐value ≤0.05 was considered significant.

## 3 RESULTS

### 3.1 History and signalment

**FIGURE 2:**
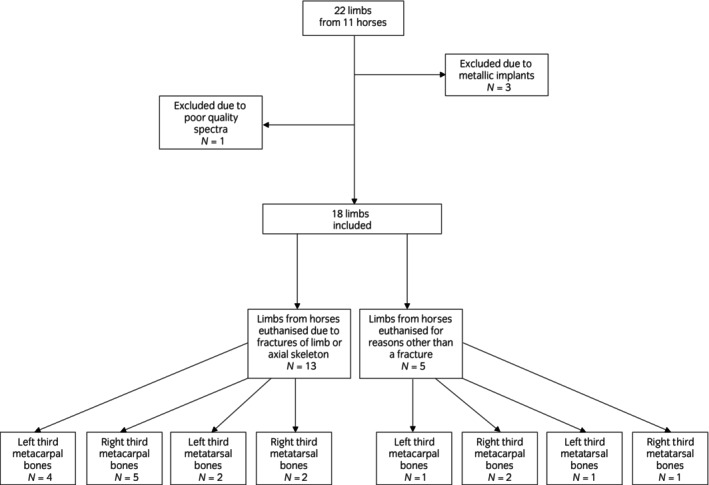
Flow chart to show the collection and inclusion of cadaver limbs in the study.

Two limbs were collected from each of the 11 horses. Of these, three were excluded following radiographic examination due to the presence of metallic implants. One was excluded due to poor quality spectrum results from the medial condyle due to a low signal to noise ratio (Figure 2). The remaining 18 limbs were from 10 National Hunt geldings with a median age of 9 years (range: 3–11 years). Figure S3 describes the limbs acquired from each horse and the cause of death in each case. Thirteen out of 18 limbs were from horses subjected to euthanasia due to fractures of the limbs or axial skeleton.

### 3.2 Bone mineral density, percentage fat content and percentage marrow space

The median value (interquartile range) for mean BMD in all included limbs was 839 (777–974) mg CaHAP/mL for the lateral condyle, 780 (729–855) mg CaHAP/mL for the medial condyle and 259 (225–280) mg CaHAP/mL for the PBM. The median value for FC (IQR) in all included limbs was 74 (68–82) % for the lateral condyle, 81 (range: 67–83) % for the medial condyle and 90 (86–92) % for the PBM. The median value for marrow space was 29 (19–34) % for the lateral condyle, 30 (23–37) % for the medial condyle and 78 (76–79) % for the PBM.

**FIGURE 3:**
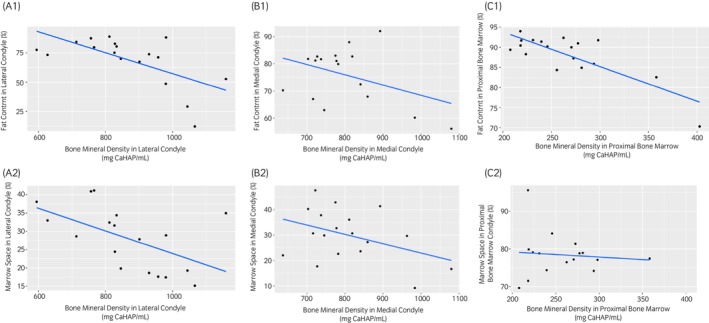
Scatter plots showing the relationship between bone mineral density and percentage fat content using MRS in the lateral condyle (1A), medial condyle (1B) and proximal bone marrow (1C) and scatter plots showing the relationship between bone mineral density and percentage marrow space from histology in the lateral condyle (2A), medial condyle (2B) and proximal bone marrow (2C).

A statistically significant moderate negative correlation was identified for mean BMD and FC (correlation coefficient = −0.6; *p* = 0.01) for the lateral condyle.^26^ Mean BMD and FC for the medial condyle showed a negligible correlation (correlation coefficient = −0.13; *p* = 0.6). A statistically significant moderate negative correlation was identified for mean BMD and FC for the PBM (correlation coefficient = −0.5; *p* = 0.03) (Figure 3).

A statistically significant moderate negative correlation was identified for mean BMD and marrow space on histology (correlation coefficient = −0.6; *p* = 0.01) for the lateral condyle. A low correlation was identified between mean BMD and marrow space for the medial condyle (correlation coefficient = −0.4; *p* = 0.2). Mean BMD and marrow space for the PBM showed a low correlation (correlation coefficient = −0.03; *p* = 0.9) (Figure 3).

### 3.3 Sclerosis grade

The sclerosis grade for MRI, CT and histology independently given by each reviewer and those condyles agreed by consensus are listed in Tables [Link], [Link], [Link]. There was agreement on sclerosis grade between the three reviewers for 32/36 condyles on MRI, 27/36 condyles on CT and 30/36 condyles on histology. Interrater agreement for MRI sclerosis grading was good to excellent (ICC = 0.9 with 95% confidence interval [CI] = 0.82–0.94), CT was moderate to good (ICC = 0.77 with 95% CI = 0.62–0.87) and histology was good to excellent (ICC = 0.86 with 95% CI = 0.76–0.92). A Kruskal–Wallis test showed that there was a statistically significant difference in median BMD between sclerosis grades in both lateral and medial condyles on MRI and CT (lateral condyle MRI [*p* = 0.001]; medial condyle MRI [0.02]; lateral condyle CT [*p* = 0.004] and medial condyle CT [*p* = 0.02]) (Table 1). There was also a statistically significant difference in FC between sclerosis groups in the lateral condyle on MRI and CT (lateral condyle MRI [*p* = 0.01]; lateral condyle CT [*p* = 0.004]) (Table 1). Furthermore, there was a statistically significant difference in percentage marrow space between sclerosis grades on histology in both the lateral (*p* = 0.006) and medial condyles (*p* = 0.009) (Table 1).

**TABLE 1:** Table showing the sclerosis grade for each limb and the median value for bone mineral density, fat content or marrow space in the lateral and medial condyles for each sclerosis grade determined by MRI, CT and histology.

Condyle	Imaging modality or histology	Sclerosis grade	Number of limbs	Median (IQR) value for mean bone mineral density (CaHAP/mL)	Median (IQR) value for fat content (%)	Median (IQR) value for marrow space (%)
Lateral	MRI	1	4	670 (619–724)	81 (76–85)	n/a
		2	10	839 (827–923)	77 (72–82)	n/a
		3	4	1055 (1028–1090)	39 (25–50)	n/a
*p*‐value				0.001*	0.01*	
Medial	MRI	1	8	736 (720–785)	82 (68–83)	n/a
		2	7	782 (757–826)	81 (76–85)	n/a
		3	3	983 (973–1031)	58 (57–59)	n/a
*p*‐value				0.02*	0.08	
Lateral	CT	1	3	713 (654–735)	84 (81–86)	n/a
		2	10	830 (815–909)	77 (73–82)	n/a
		3	5	1044 (980–1067)	49 (29–53)	n/a
*p*‐value				0.004*	0.004*	
Medial	CT	1	6	736 (723–767)	82 (81–83)	n/a
		2	9	782 (737–841)	80 (70–82)	n/a
		3	3	983 (973–1031)	58 (57–59)	n/a
*p*‐value				0.02*	0.06	
Lateral	Histology	1	2	n/a	n/a	35 (31–38)
		2	11	n/a	n/a	32 (28–35)
		3	5	n/a	n/a	18 (17–19)
*p*‐value						0.006*
Medial	Histology	1	4	n/a	n/a	38 (35–42)
		2	11	n/a	n/a	30 (25–34)
		3	3	n/a	n/a	17 (13–17)
*p*‐value						0.009*

*Note*: Statistically significant differences between sclerosis grades calculated using Kruskal–Wallis tests are indicated by an asterisk (*). A *p*‐value ≤0.05 was considered significant.

## 4 DISCUSSION

The ^1^H spectra were successfully quantified at three different ROIs within the MC/MTIII to calculate bone marrow FC in these sites showing that MRS is a viable imaging modality for use in horses.

Our results suggest that FC decreases with increasing BMD in the trained racehorse however this relationship only exists in the lateral condyle and PBM in this study population. Several studies have previously identified an increase in BMD measured using CT, in the condyles of MC/MTIII in trained racehorses compared with untrained racehorses.^2^, ^27^, ^28^ The adaptive modelling process leads to bone deposition on the surface of the trabeculae, reduction in the size of the fat‐filled marrow spaces and a reduction in bone marrow adiposity.^29^ Although not as widely reported as in the condyles, an increase in volumetric BMD in response to training has been described in the diaphysis of MCIII of 2‐year‐old Thoroughbreds, however the difference in BMD of the diaphysis between trained (1117 mg/cm^3^) and untrained (1096 mg/cm^3^) horses was not as great as in the condyles (trained: 1140 mg/cm^3^; untrained 1110 mg/cm^3^).^30^ Two main outliers were identified in the scatter plot looking at the relationship between BMD and FC in the lateral condyle. These condyles showed a very low FC and a high BMD. One of these limbs showed evidence of a lateral condylar fracture of MCIII and the other had a transverse fracture of MCIII originating from the lateral condyle. One study looking at MCIII condylar fractures reported that these fractures occurred at a site of focal increase in BMD which may explain the increased BMD whilst the very low FC may be due to an increase in trabecular thickness in this region.^31^ Future research should include a larger population of limbs with lateral condylar fractures to investigate this relationship further. A negligible correlation was found between BMD and FC in the medial condyles. This is likely due to the low power in this pilot study and warrants further investigation in a larger group of limbs.

Marrow space was calculated as a proxy for FC, presuming that the larger the marrow space in a particular section, the higher the FC. There was a negative correlation between marrow space and BMD in the lateral condyle and a difference in marrow space noted between sclerosis grades suggesting that marrow space is a good proxy for FC in the lateral condyle. However, a low correlation existed between marrow space and BMD in the medial condyle and PBM. This finding in the medial condyle is similar to the negligible correlation found when comparing FC calculated from MRS with BMD and is likely due to the low sample size. When comparing FC calculated from MRS with BMD in the PBM there was a moderate negative correlation found which differs from the low correlation found for marrow space. The PBM region of bone has a large area of marrow space compared with the condyles and this finding suggests that calculating marrow space may not detect small changes in FC unlike when calculating FC on MRS. Future work could include histology sections stained with Oil red O as this identifies neutral lipids and fatty acids and may allow for more accurate quantification of FC.^32^

Sclerosis can be defined as densification of bone.^33^ It is therefore not unexpected that there was a significant difference in BMD, FC and marrow space between sclerosis grades determined by MRI, CT and histology. Further post hoc testing could be carried out in future studies with a larger sample size to determine which specific grades differ. It should be noted that although the sclerosis grading scale was from 0 (normal) to 3 (severe), no condyles were found to be entirely normal. All of these cadaver limbs were collected from Thoroughbred racehorses in full work. Previous work has shown that fatigue damage in the subchondral bone of the distal metacarpal condyle is extremely prevalent in this population of horses and that it tends to accumulate throughout a horse's racing career.^29^, ^30^, ^33^ In one study subchondral bone sclerosis was found to be suggestive of microdamage. All limbs included in this study were from racing Thoroughbreds and all limbs showed some evidence of sclerosis.^33^

As previously discussed, exercise has been shown to be inversely correlated with bone marrow FC in the lumbar vertebrae of humans in several studies, although the same relationship appears to be less consistent in the proximal femur and tibia of humans.^15^, ^16^, ^34^ In one of these studies at least 2 h of weekly exercise was required to cause a consistent reduction in bone marrow FC compared with lower levels of exercise whilst the other studies assessed this relationship in professional footballers and regular long‐distance runners.^34^ Exercise has also been found to be inversely correlated with bone marrow FC in the femur and tibia of mouse models.^35^, ^36^ A dynamic relationship is known to exist between osteoblasts and adipocytes within bone marrow, and mechanical loading has been shown to inhibit adipocyte differentiation and promote osteoblastogenesis in these models.^37^, ^38^ This might be a further explanation for the inverse relationship identified between bone marrow FC and BMD in horses. Future work might include a resting age and sex matched control group to determine if a similar relationship exists with exercise and at what level of exercise changes in bone marrow FC become evident. An assessment of bone marrow FC during different stages of racehorse training might allow further understanding of the equine bone metabolism in response to exercise.

Several studies have identified age‐related changes in BMD and subchondral bone microstructure in horses.^33^, ^39^, ^40^ The relationship between age and BMD, FC or marrow space was not investigated in this study due to the small sample size. In humans, studies have found that bone marrow adiposity increases and BMD decreases with increasing age.^41^, ^42^ Differences between males and females have also been identified.^42^, ^43^ Further investigation with a larger population of horses would be required to determine if trends exist for age and sex in horses.

The major limitation of this study was the small sample size included at convenience. This was partly due to there being no pre‐existing data on proton MRS in equine bone to support effect size. The data from our pilot study could therefore be used to guide larger studies in future. As this was an observational study to determine the feasibility of this novel technique in equine bone, no matched controls were included. Future studies to investigate differences between horses with fractures and those without should aim to recruit age and sex‐matched controls from the same yard with similar training history and without evidence of a fracture. Additionally, the population included only geldings. Studies in humans have found bone marrow adiposity differences between males and females at different stages of life.^43^, ^44^ Although it is unknown whether this is also true for horses, inclusion of mares in the study would have been a better reflection of the general population of racehorses in training as well as providing the potential for intersex comparisons. This pilot study was performed ex vivo to determine if MRS is possible in equine bone. Further resources and potential risks would be associated with performing this in vivo due to the requirement for general anaesthesia. Limbs were frozen prior to imaging for practical reasons and limb temperature was not directly measured prior to MRI. In order to assess the impact of this, in the study design stage, one fresh and one freeze–thawed limb underwent preliminary testing and both produced similar MRS signal. Several cadaver studies have found no difference in MR image quality in freeze–thawed equine bone.^35^, ^36^, ^37^ Furthermore, a human cortical bone study found that up to 6 freeze‐thaw cycles had a negligible impact on MRS properties.^38^ A further limitation of this pilot study was a mild discrepancy between the MRS and CT ROIs used to calculate percentage FC and BMD and the histology slices used to calculate percentage marrow space. Although every effort was made to match the histology slices with the MRS and CT ROIs as closely as possible, there is an unavoidable difference in the scale of the two techniques when comparing a 2 mm histology slice with a 1 cm^3^ ROI. Future studies could consider reducing the size of the MRS ROI, however this would reduce the signal‐to‐noise ratio and may make quantification of percentage FC more challenging.

One limb was excluded due to poor quality spectrum results from the medial condyle due to a low signal to noise ratio. This may have been due to poor shimming. The use of manual shimming could be investigated for use in difficult cases in the future although this can be user‐dependent and time consuming.

Future research using MRS could aim to investigate the relationship between bone marrow adiposity and BMD in a larger group of horses with and without stress fractures either in MC/MTIII or at other common stress fracture sites. The preliminary data in the current study can be used to carry out power calculations for future studies. Several human studies have established that higher bone marrow FC is associated with lower BMD and increased prevalence of vertebral fracture.^11^, ^45^ However, these studies were performed in a population with osteoporosis or other endocrine diseases. Osteoporosis has a very different aetiopathogenesis to stress fracture development in the horse and to date no studies have been performed looking at bone marrow FC in human athletes with stress fractures. The spectra could be further analysed in future studies to determine the proportion of unsaturated and saturated fat within the bone marrow as a human study found that bone marrow fat composition can differ between women with and without fragility fractures.^11^

In conclusion, proton MRS can be performed in the equine MC/MTIII and has the potential to provide further information on the role of marrow adipose tissue in bone health. This pilot feasibility study has confirmed that further studies designed to assess the use of MRS as a screening tool to predict fracture risk in racehorses are warranted.

## FUNDING INFORMATION

This study was supported by the Horseracing Betting Levy Board.

## CONFLICT OF INTEREST STATEMENT

The authors declare no conflicts of interest.

## AUTHOR CONTRIBUTIONS

**Charlotte Hewitt‐Dedman:** Data curation; formal analysis; investigation; methodology; project administration; resources; writing – original draft; writing – review and editing. **Lucy Kershaw:** Data curation; methodology; resources; software; supervision; writing – review and editing. **Tobias Schwarz:** Data curation; methodology; resources; software; writing – review and editing. **Jorge Del‐Pozo:** Data curation; methodology; resources; software; supervision; writing – review and editing. **Juliet Duncan:** Data curation; formal analysis; supervision; writing – review and editing. **Carola R. Daniel:** Data curation; investigation; resources; software; writing – review and editing. **Eugenio Cillán‐García:** Data curation; resources; writing – review and editing. **Maria Chiara Pressanto:** Data curation; resources; writing – review and editing. **Sarah Taylor:** Conceptualization; data curation; funding acquisition; investigation; methodology; project administration; supervision; writing – review and editing.

## DATA INTEGRITY STATEMENT

C. L. Hewitt‐Dedman confirms that she had full access to all the data in the study and takes responsibility for the integrity of the data and the accuracy of the data analysis.

## ETHICAL ANIMAL RESEARCH

University of Edinburgh Ethics Committee VERC 140.19.

## INFORMED CONSENT

All included horses had informed consent from owner and trainer.

### PEER REVIEW

The peer review history for this article is available at https://www.webofscience.com/api/gateway/wos/peer-review/10.1111/evj.14086.

## Supporting information


**Figure S1.** T1‐weighted frontal MR images of the metacarpo/tarsophalangeal joint demonstrating the sclerosis grading scale. Lateral is to the left of each image. Image A shows an example of mild sclerosis in both condyles (grade 1), image B shows moderate sclerosis (grade 2) in both condyles and image C shows severe sclerosis (grade 3) in both condyles.

**Figure S2.** Frontal CT images of the metacarpo/tarsophalangeal joint demonstrating the sclerosis grading scale. Lateral is to the left of each image. Image A shows an example of mild sclerosis in both condyles (grade 1), image B shows moderate sclerosis (grade 2) in both condyles and image C shows severe sclerosis (grade 3) in both condyles.

**Figure S3.** Histology sections taken from the lateral parasagittal groove of the distal metacarpus/tarsus showing an example of the sclerosis grading scale. Image A shows an example of mild sclerosis (grade 1), image B shows moderate sclerosis (grade 2) and image C shows severe sclerosis (grade 3).

**Table S1.** Pulse sequence parameters for high‐field 3 T MRI system.

**Table S2.** Parameters for 64‐slice helical CT scanner.

**Table S3.** Table showing a description of the limbs acquired and the cause of death for each horse.

**Table S4.** Table showing sclerosis grading on MRI for each condyle in each limb for each reviewer and if no absolute agreement, then the consensus grade.

**Table S5.** Table showing sclerosis grading on CT for each condyle in each limb for each reviewer and if no absolute agreement, then the consensus grade.

**Table S6.** Table showing sclerosis grading on histology for each condyle in each limb for each reviewer and if no absolute agreement, then the consensus grade.

## Data Availability

The data that support the findings of this study are openly available in University of Edinburgh at https://datashare.ed.ac.uk/handle/10283/8762.
