# Supplementary material for: Preliminary study of proton magnetic resonance spectroscopy to assess bone marrow adiposity in the third metacarpus or metatarsus in Thoroughbred racehorses
Source: Equine Vet J. 2024 May 3;57(2):471–9. doi: 10.1111/evj.14086 (PMC11807939; doi:10.1111/evj.14086)

Equine Veterinary Journal BEVA

Figure S1: T1-weighted frontal MR images of the metacarpo/tarsophalangeal joint demonstrating the sclerosis grading scale. Lateral is to the left of each image. Image A shows an example of mild sclerosis in both condyles (grade 1), image B shows moderate sclerosis (grade 2) in both condyles and image C shows severe sclerosis (grade 3) in both condyles.

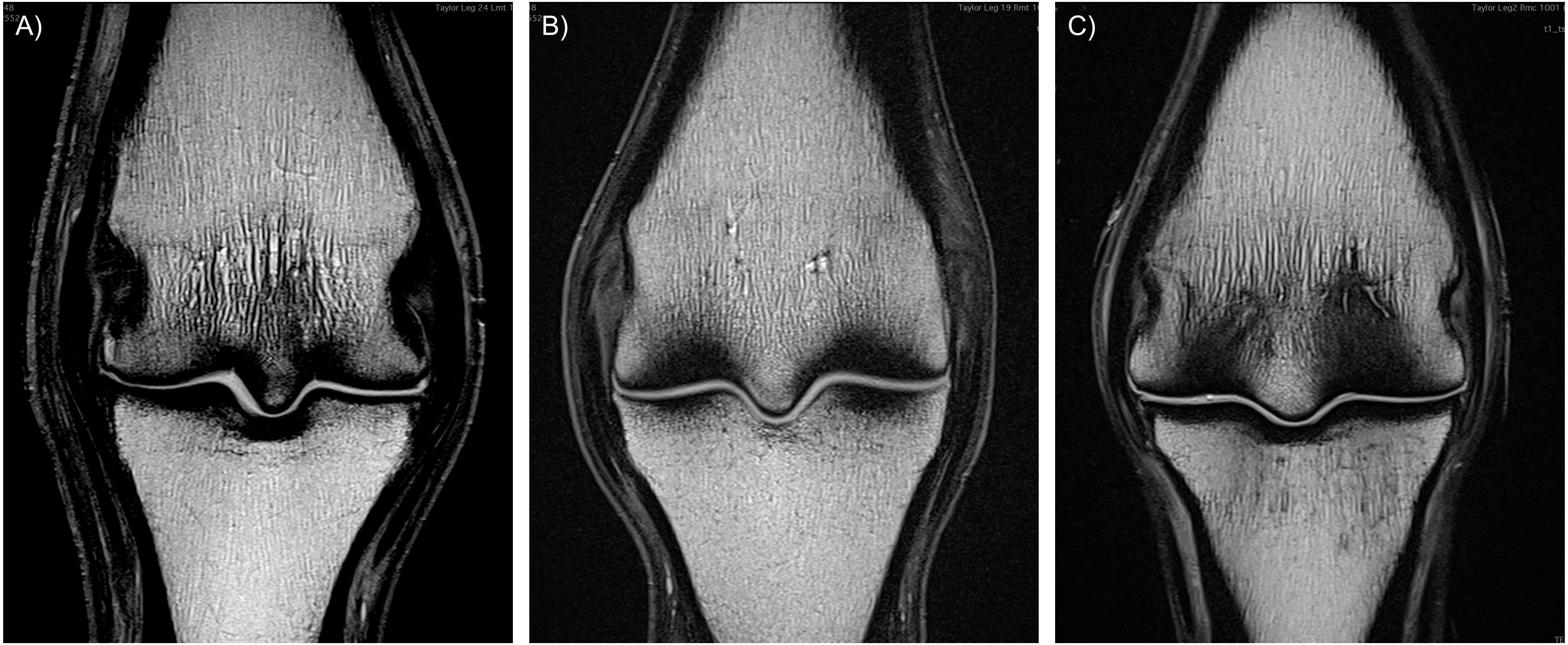

Supplement: Supplementary file 1 — Figure S1. T1‐weighted frontal MR images of the metacarpo/tarsophalangeal joint demonstrating the sclerosis grading scale. Lateral is to the left of each image. Image A shows an example of mild sclerosis in both condyles (grade 1), image B shows moderate sclerosis (grade 2) in both condyles and image C shows severe sclerosis (grade 3) in both condyles. [file EVJ-57-471-s005.pdf]
