# Supplementary material for: Preliminary study of proton magnetic resonance spectroscopy to assess bone marrow adiposity in the third metacarpus or metatarsus in Thoroughbred racehorses
Source: Equine Vet J. 2024 May 3;57(2):471–9. doi: 10.1111/evj.14086 (PMC11807939; doi:10.1111/evj.14086)

Equine Veterinary Journal BEVA

Figure S3: Histology sections taken from the lateral parasagittal groove of the distal metacarpus/tarsus showing an example of the sclerosis grading scale. Image A shows an example of mild sclerosis (grade 1), image B shows moderate sclerosis (grade 2) and image C shows severe sclerosis (grade 3).

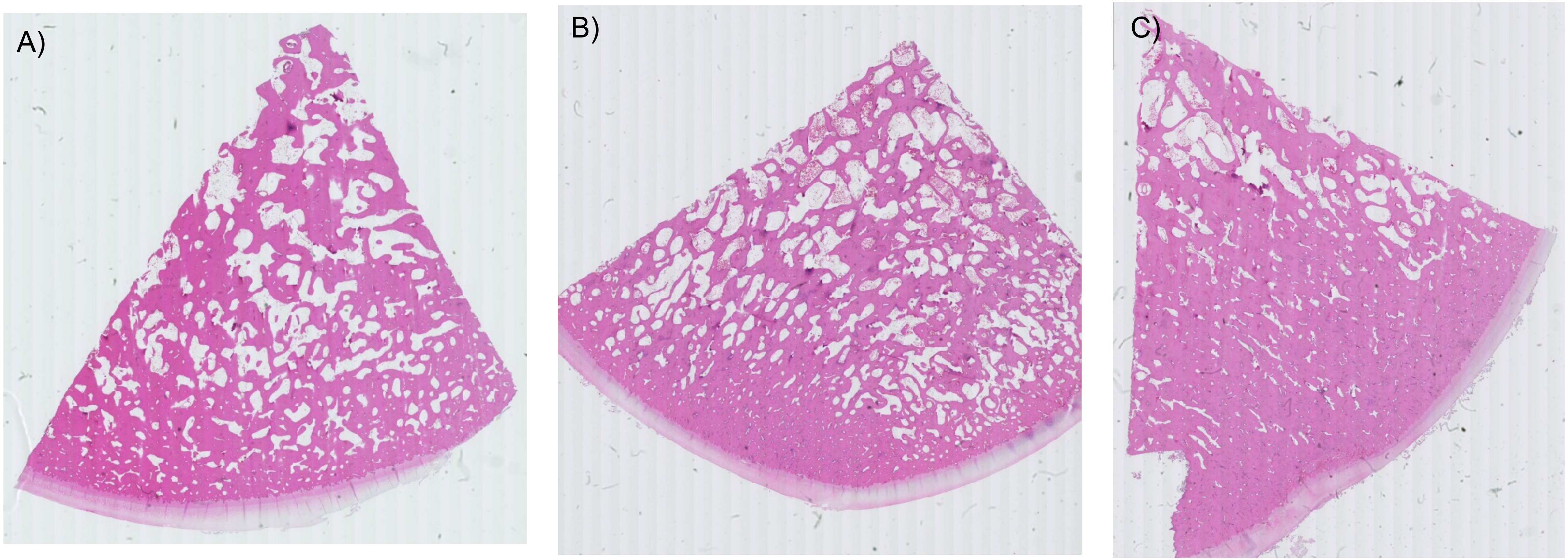

Supplement: Supplementary file 3 — Figure S3. Histology sections taken from the lateral parasagittal groove of the distal metacarpus/tarsus showing an example of the sclerosis grading scale. Image A shows an example of mild sclerosis (grade 1), image B shows moderate sclerosis (grade 2) and image C shows severe sclerosis (grade 3). [file EVJ-57-471-s003.pdf]
