# Supplementary material for: Preliminary study of proton magnetic resonance spectroscopy to assess bone marrow adiposity in the third metacarpus or metatarsus in Thoroughbred racehorses
Source: Equine Vet J. 2024 May 3;57(2):471–9. doi: 10.1111/evj.14086 (PMC11807939; doi:10.1111/evj.14086)
Supplement: Supplementary file 4 — Table S1. Pulse sequence parameters for high‐field 3 T MRI system. [file EVJ-57-471-s002.pdf]

Equine Veterinary Journal BEVA

Table S1: Pulse sequence parameters for high-field 3T MRI system.

Pulse Sequence	Orientation	TE (ms)	TR (ms)	ST(mm)	Number of Averages
T1W TSE	Sagittal<br>Frontal<br>Transverse	20	1660	1.6	2
STEAM	n/a	20	4000	n/a	70

TSE: Turbo Spin Echo; STEAM: Stimulated Acquisition Mode; TE: echo time; TR: repetition time; ST: slice thickness
