# Supplementary material for: Preliminary study of proton magnetic resonance spectroscopy to assess bone marrow adiposity in the third metacarpus or metatarsus in Thoroughbred racehorses
Source: Equine Vet J. 2024 May 3;57(2):471–9. doi: 10.1111/evj.14086 (PMC11807939; doi:10.1111/evj.14086)
Supplement: Supplementary file 5 — Table S2. Parameters for 64‐slice helical CT scanner. [file EVJ-57-471-s007.pdf]

Equine Veterinary Journal BEVA

**Table S2:** Parameters for 64-slice helical CT scanner.

| kVp | mAs | ST (mm) | Matrix Size | Pixel Spacing (mm) | Table Height (cm) |
|---|---|---|---|---|---|
| 120 | 117 | 0.4 | 512 x 512 | 0.3 | 125 |

*kVp: peak kilovoltage; mAs: milliampere-seconds; ST: slice thickness*
