# Supplementary material for: Preliminary study of proton magnetic resonance spectroscopy to assess bone marrow adiposity in the third metacarpus or metatarsus in Thoroughbred racehorses
Source: Equine Vet J. 2024 May 3;57(2):471–9. doi: 10.1111/evj.14086 (PMC11807939; doi:10.1111/evj.14086)
Supplement: Supplementary file 6 — Table S3. Table showing a description of the limbs acquired and the cause of death for each horse. [file EVJ-57-471-s001.pdf]

Equine Veterinary Journal BEVA

Table S3: Table showing a description of the limbs acquired and the cause of death for each horse.

Horse No.	Cause of Death	Limb No.	Metacarpus or Metatarsus	Left or Right
1001	Superficial digital flexor tendon rupture	1	Metacarpus	Right
1002	Acute occipital fracture with dural laceration	2	Metacarpus	Left
1002		3	Metacarpus	Right
1003	Lumbar fracture	4	Metacarpus	Right
1003		5	Metacarpus	Left
1004	Transverse metacarpal fracture originating from lateral condyle of the third metacarpus	6	Metacarpus	Right
1005	Humeral fracture	7	Metacarpus	Left
1005		8	Metacarpus	Right
1006	Open, displaced fracture of the lateral condyle of the third metacarpus fracture	9	Metacarpus	Left
1006		10	Metacarpus	Right
1007	Left carotid artery tear and severe mediastinal haemorrhage	11	Metatarsus	Left
1007		12	Metatarsus	Right
1008	Humeral fracture	13	Metacarpus	Left
1008		14	Metacarpus	Right
1009	Severely comminuted proximal phalanx fracture	15	Metatarsus	Left
1009		16	Metatarsus	Right
1010	Lumbar fracture	17	Metatarsus	Left
1010		18	Metatarsus	Right
