# Supplementary material for: Preliminary study of proton magnetic resonance spectroscopy to assess bone marrow adiposity in the third metacarpus or metatarsus in Thoroughbred racehorses
Source: Equine Vet J. 2024 May 3;57(2):471–9. doi: 10.1111/evj.14086 (PMC11807939; doi:10.1111/evj.14086)
Supplement: Supplementary file 8 — Table S5. Table showing sclerosis grading on CT for each condyle in each limb for each reviewer and if no absolute agreement, then the consensus grade. [file EVJ-57-471-s004.pdf]

Equine Veterinary Journal BEVA

Table S5: Table showing sclerosis grading on CT for each condyle in each limb for each reviewer and if no absolute agreement, then the consensus grade.

	Sclerosis Grade							
	Reviewer 1		Reviewer 2		Reviewer 3		Consensus	
Limb No.	Lateral Condyle (0-3)	Medial Condyle (0-3)	Lateral Condyle (0-3)	Medial Condyle (0-3)	Lateral Condyle (0-3)	Medial Condyle (0-3)	Lateral Condyle (0-3)	Medial Condyle (0-3)
1	2	3	2	3	2	3	Agreement	
2	2	1	2	1	2	1	Agreement	
3	2	1	3	1	3	1	3	Agreement
4	2	1	2	1	2	1	Agreement	
5	2	1	3	1	3	1	3	Agreement
6	3	2	3	2	3	2	Agreement	
7	2	2	1	1	1	2	1	2
8	2	2	2	2	2	2	Agreement	
9	3	3	3	3	3	3	Agreement	
10	3	3	2	3	3	3	3	Agreement
11	2	2	1	1	2	2	Agreement	
12	2	2	1	1	2	2	Agreement	
13	1	2	1	2	1	2	Agreement	
14	2	2	1	2	2	2	2	Agreement
15	2	2	2	1	2	2	Agreement	2
16	2	1	2	1	2	1	Agreement	
17	1	1	1	1	1	1	Agreement	
18	2	2	1	1	2	2	2	2
